# Assessing the effectiveness of road safety measures in Barcelona (2013-2018)

**DOI:** 10.1016/j.heliyon.2023.e23063

**Published:** 2023-11-29

**Authors:** Lluís Bermúdez, Isabel Morillo

**Affiliations:** a Department of Economics, Financial and Actuarial Mathematics, University of Barcelona, Spain; b Riskcenter-IREA, University of Barcelona, Spain

**Keywords:** 62J12, 62P99, Urban mobility, Logistic regression, Traffic crash, Crash severity, Policy implications

## Abstract

**Introduction:**

This article aims to determine the effectiveness and extent of measures taken to decrease the severity of traffic crashes in Barcelona from 2013 to 2018. This will be achieved through an analysis of the traffic crash data.

**Method:**

Our approach involves the use of binary logistic regression models. We rely on the traffic crash dataset from 2010-2019 available in the Open Data Barcelona platform.

**Results:**

The outcomes obtained from the suggested models are contrasted with the strategies outlined in the Local Road Safety Plan 2013-2018 to minimize the severity of crashes. Effective preventive actions were identified, such as road safety educational programs, creating calm zones, enhancing pedestrian crossings, or expanding bicycle lanes. However, certain measures were found to be ineffective or their impact remained uncertain.

**Conclusions:**

Our findings indicate that the measures implemented in Barcelona may have participated in and influenced the decrease in the severity of traffic incidents over the past decade. Notably, fatalities have decreased more than severe injuries. More attention should be given to less effective measures such as speed controls and drug/alcohol testing.

## 1 Introduction

Reducing traffic crashes is a priority for European cities, including Barcelona. In recent years, the Barcelona City Council has approved several local plans aimed at improving road safety and urban mobility. The Local Road Safety Plan (LRSP) for the period of 2013-2018 [1] included 24 measures and 80 specific actions with the goal of reducing traffic fatalities and serious injuries by 20% by 2018 compared to 2012 levels. These targets were in line with those established by the European Road Safety Action Programme 2011-2020 [2], which aimed to reduce deaths and serious injuries by 50% and 40% respectively by 2020 compared to 2010. In Barcelona, the number of fatalities in 2018 decreased by 46% compared to 2012, but the number of serious injuries only decreased by 9%, falling short of the 20% target.

The study of traffic crashes may help to assess the efficacy of measures implemented by policymakers in reducing the frequency and severity of such crashes. Researchers have examined various factors that contribute to road traffic crashes and assessed the impact of countermeasures in addressing them. For example, [3] analyzed the severity of crashes on the German Autobahn to evaluate the effectiveness of the 2011 German traffic safety program. Their empirical findings indicated strong support for the implemented measures, suggesting that they should be continued. Implementing intelligent traffic control systems, preventing lane departures from the roadway, and enhancing safety measures for pedestrians and motorcyclists can effectively decrease the severity of crashes. [4] evaluated the severity of traffic crashes in China from a macro perspective and recommended effective measures to reduce fatalities, such as safeguarding vulnerable road users, imposing speed limits, discouraging nighttime driving, widening safety margins, and enhancing road infrastructure design. [5] explored strategies for minimizing traffic crashes involving young individuals, proposing the implementation of early awareness campaigns, starting as early as kindergarten to foster responsible traffic behaviors from an early age. After these initial campaigns, there should be subsequent broader initiatives aimed at increasing the age range of the target group. Similar examples can be found in [6], [7], and [8].

The purpose of this paper is to assess the effectiveness of measures implemented in the LRSP 2013-2018 to reduce fatalities and serious injuries in traffic crashes in Barcelona. The technical actions planned in the LRSP 2013-2018 focused on preventive and corrective actions, as well as educating people on road safety. The city council's local road safety plans serve as the primary tool for enhancing road safety, reducing crashes, and regulating traffic flow within the city. From 2004 to 2011, Barcelona witnessed a consistent decline in both the overall number of traffic-related incidents and the severity of resulting injuries. However, in 2012, a shift in this trend became evident, highlighting the need to reevaluate road safety policies. In 2012, crashes involving pedestrians, motorcycles and bicycles accounted for 81% of incidents. Therefore, it became imperative to update the road safety policies while continuing with ongoing projects and identifying new priority areas for intervention. This gave rise to the Barcelona LRSP 2013-2018, aimed at continuing efforts to reduce the number of serious injuries and fatalities. In the LRSP 2013-2018, the priority areas for intervention were incidents involving running down, casualties with motorcycles, and vulnerable groups such as cyclists, pedestrians, and moped users. The Local Road Safety Plan in Barcelona 2019-2022 [9] reported on the progress made in implementing the LRSP 2013-2018: out of the 80 technical measures outlined in the plan, 80% had been completed, 15% were in the development phase, and 5% had not been implemented due to certain impediments. Table 1 presents an overview of the actions carried out under the LRSP 2013-18, along with their respective implementation schedules.

**Table 1:** Summary of time schedule and road safety measures implemented within the LRSP 2013-2018.

*Corrective actions*	
Installation of speed cameras at high-accident intersections	2014-2018
Augmentation of photo-red cameras to deter red-light violations	2014-2018
Enhancement of traffic light technology and alignment	2015-2018
Fine-tuning of traffic light timings to enhance pedestrian and cyclist mobility	2013-2018
Expansion and enhancement of pedestrian zones	2013-2018
Enhancement of pedestrian crossings through signaling and traffic light upgrades	2013-2018
Enforcement of the 30 km/h zones, including rigorous speed monitoring	2013-2018
Implementation of a strategic cycling plan, including the expansion of cycling lanes and parking facilities	2016-2018
Prohibition of bicycles on sidewalks narrower than 3 meters in width	2014-2018
Improvement of intersection configurations within urban areas	2013-2018
*Preventive actions*	
Emphasizing the control of high-risk driving offenses	2013-2018
Maintaining or increasing alcohol and drug testing with special focus on motorcyclists	2013-2018
Conducting macro-level alcohol and drug controls in specified areas and during certain times of the year	2013-2018
Enhancing mobile speed enforcement campaigns and augmenting speed limit signage	2013-2018
Vigorous enforcement of approved bicycle traffic regulations	2013-2018
Inspection of child restraint systems, seat belts, and helmets	2013-2018
Implementing static and dynamic surveillance campaigns at intersections targeted at motorcyclists	2013-2018
Conducting specialized speed and MOT checks for motorcyclists	2016-2018
Substituting penalties for severe infractions with educational training courses	2015-2018
Initiating campaigns to combat driver distractions, including mobile phone and GPS usage	2016-2018
Initiating efforts to enhance safety in bus and taxi lanes	2016-2018
Promoting safety in school transportation through dedicated campaigns	2016-2018
Employing dynamic enforcement tools for detecting moving violations	2016-2018
Enacting regulations to enhance the safety of personal mobility vehicle users, pedestrians, and cyclists	2017-2018
Delivering training activities tailored for various educational stages of minors	2013-2018
Organizing safe mobility sessions for elderly individuals	2014-2018
Providing road safety tools through training sessions for company employees	2014-2018
Distributing driving tips to registered motorcyclists through informational channels	2015-2018

To evaluate the impact of the measures introduced in the LRSP 2013-2018 aimed at decreasing fatalities and severe injuries in Barcelona, we have employed a dataset obtained from the local police department's traffic database. This dataset comprises information on traffic crashes involving victims spanning from 2010 to 2019. Our primary objective is to determine whether the implementation of these measures has resulted in a decline in both fatalities and severe injuries over time. To facilitate our analysis, we have categorized the years during which these crashes occurred into three distinct periods. The first period, from 2010 to 2012, serves as our reference or control period, representing the time before the measures were put into effect. The second period, spanning from 2013 to 2016, corresponds to the phase during which the majority of the measures were actively implemented. Finally, the third period, from 2017 to 2019, signifies a stage where the implementation of the plan can be considered nearly completed. According to local police [9], no significant alterations in traffic conditions that would impact the severity of crashes in Barcelona were identified during the LRSP 2013-2018 period. Consequently, it is reasonable to assume that the alterations in crash severity observed across these three timeframes can be mainly attributed to the modifications introduced by the Plan. Additionally, within a regression modeling framework, we evaluate the effect of the year period in which the crash occurred into crash-related injuries' severity, controlling for other different risk factors including type and cause of crash, number and type of vehicles involved, day types, and type of road where crash occurred.

The analysis of crash-injury severity in literature is highly developed. For an in-depth examination of the modeling frameworks employed, please refer to [10], [11] and [12]. Logistic regression is commonly used to represent injury severity in binary form. In [13], crash severity is a dichotomous variable with two categories, fatal and non-fatal. [14] used a logistic regression model for serious and fatal injuries to drivers involved in crashes between two passenger vehicles. In [15] used a step-wise logistic regression model to identify risk factors affecting injury severity for multiple vehicle traffic crashes in Hong Kong, categorizing injury severity into “fatal/serious” and “slight”. [16] and [17] analyzed the risk of pedestrian injury and fatality in traffic crashes and applied the model considering the two categories of severely injured/dying versus minor/no injured severity.

In our study, we employed this latter binary classification approach to categorize the severity of traffic crash injuries into two groups: fatal/serious versus non-serious (minor or slight). We then applied multiple binary logistic regression models to assess how various risk factors influence the severity of injuries resulting from crashes. First, as previously mentioned, we plan to assess the effectiveness of LRSP measures in reducing fatal and seriously injured victims during the entire examined period. Our second aim is to determine whether the impact of other risk factors on crash severity has undergone any significant changes and to evaluate the impact of the implemented measures on this phenomenon. To achieve this, we have tailored a specific logistic regression model for the 2010-2012 reference subperiod and a separate one for the concluding subperiod of 2017-2019. Finally, we have employed various logistic regression models for different severity levels to evaluate the impact of traffic measures on fatalities and serious injuries separately. This study provides a discussion and summary of the results of these analyses.

## 2 Data and methods

The dataset on traffic crashes during the period of 2010-2019 in Barcelona was sourced from the local police department's traffic accident database. This database is available to the public through Open Data BCN, which enables access to information generated or maintained by public entities. For each year, there are five interlinked datasets with a record code that provide information on the crashes, the mediate cause of the crash, the type of crash, as well as the vehicles and victims involved in the crash.

The dataset contains a total of 96,728 motor-vehicle crashes reported by police, which occurred between 1 January 2010 and 31 December 2019. To consolidate the information and ensure that each crash had only one entry in the database, some modifications were made to each dataset (e.g. eliminating redundant variables and creating dummy variables for crashes with multiple observations). After merging the datasets, the final database included 87,823 traffic crashes that involved victims during the study period. Traffic crashes that did not involve victims were excluded for two reasons: firstly, minor crashes often do not require police presence and therefore the sample of these types of crashes would be biased; secondly, the focus of the study is on crashes resulting in fatal, serious or non-serious injuries. The victims are divided into three categories: fatal (deaths), seriously injured (hospitalized for more than 24 hours), and non-serious injured (treated at the scene of the incident, in hospital emergency services, or hospitalized for less than 24 hours).

The variables used in the analysis are shown in Table 3. First, assessing road safety in urban regions involves examining the severity of injuries sustained by victims of traffic crashes, which is a crucial indicator. In this study, we focus on injury severity as the dependent variable, which can be assessed through various metrics available in the database. As we stated in the introduction, we have employed a dichotomous variable *Severity*, which separates crashes into two categories: those with either fatal or serious injuries and those with only non-serious injuries. Typically, as used here, crash severity is assessed by measuring the level of injury suffered by the most seriously injured vehicle occupant [18].

Second, as previously discussed in the introduction, it is reasonable to assume that observing the *Period* during which the crash occurred enables us to assess the effectiveness of the measures implemented to reduce the severity of crashes. For this purpose, we have categorized the *Period* variable into three distinct phases: the first (2010-2012) as a reference or control period before measures were implemented, the second (2013-2016) as the active implementation phase of most measures, and the third (2017-2019) as a stage where implementation was nearing completion. The evolution of injury severity over time for these three periods is presented in Table 2, which shows a reduction in the percentage of crashes with victims resulting in fatalities and serious injuries. However, there is a far greater reduction in crashes with fatalities than those resulting in serious injuries.

**Table 2:** Evolution of the severity of injuries 2010-2019. The percentages of change as compared to 2010-2012 period are shown in parentheses.

Variable	Period					
	2010-2012		2013-2016		2017-2019	
	Total	%	Total	% (Δ%)	Total	% (Δ%)
non-serious	23,721	96.94	34,668	97.29 (0.37)	27,040	97.54 (0.62)
serious	656	2.68	860	2.41 (-9.97)	626	2.26 (-15.76)
fatalities	93	0.38	104	0.29 (-23.20)	55	0.20 (-47.80)

Finally, numerous additional factors may impact the severity of a traffic crash. To comprehensively evaluate the LRSP 2013-2018's effectiveness, it is essential controlling for other relevant factors that can either mitigate or exacerbate injury severity in such incidents. Many studies have been conducted to identify the factors that impact the severity of crashes. For instance, [19] analyzed the risk factors in urban road traffic crashes that had the most significant impact on users who were fatally, severely, and mildly injured. In our study, we have employed a set of controlling factors that reflect the characteristics of the crash, such as the time and site of the crash, vehicle-related characteristics, related causes and type of crash.

Leaving aside the *Period* variable, the time of the crash contains *Daytype* (distinguishing between working day and weekend day) and *Daypart* (shifts of the day: morning, afternoon and evening-night). The crash site (*Via*) refers to the type of road where the incident took place, which is categorized into street, avenue or fast lane. The study classified the factors *Period*, *Daytype*, *Daypart* and *Via* into several categories, with the first category serving as the reference. The variables related to vehicles include the number (*Vehicles*) and type of vehicles involved, such as *Bicycle*, *Twowheel*, *Heavy* and *Light*. *Vehicles* is a dichotomous variable with two categories (crashes with more than one vehicle involved and other). Regarding the type of vehicles involved, we have defined four dichotomous variables that indicate the presence or absence of each type of vehicle in the crash. Related causes of the crash include variables such as *Pedestrian* (when the crash is due to the behavior of a pedestrian), *Bloodalcohol* (when an excessive blood alcohol concentration level is detected by the local police), *Speed* (when excessive speed, driving faster than the posted limit, has been considered by local police as the cause of the crash) and *Roadcondition* (when the state of the road is the registered cause of the crash). In this case, we have also defined four dichotomous variables that indicate the presence or absence of each cause according to the police report. Please note that a single crash may include more than one type of vehicle involved or related cause. Finally, crash type variables include *Rundown* (when a person has been hit by the car and as a result is lying down on the ground), *Twowheelfall* (a crash implying a fall from a two-wheel vehicle), *Collision* (when a vehicle collides with another vehicle), and *Shock* (when a vehicle collides with a stationary object). As the above variables, they were defined as dichotomous variables indicating the presence or absence of each type of crash. These potential influencing factors have been widely used in several studies [20], [21], [19], [15], [4], [22].

For descriptive purposes, the data presented in Table 4 indicates that the most severe crashes were more likely to occur on weekends and during nighttime hours. Furthermore, crashes leading to fatal injuries were more frequent on fast lanes than in regular streets and avenues (streets with two directions of circulation or main arteries of the city with more than 2 km in length), which contrasts with the pattern observed for crashes with serious injuries. Additionally, crashes involving a single vehicle were found to have a higher incidence of serious and fatal injuries. These findings align with earlier research on crash severity in urban locations [20], [19], [15], [16], [23]. Looking at the changes between the periods of 2010-2012 and 2017-2019, we can observe a significant decrease in fatalities and serious injuries during working days and the morning shift, and also on regular streets. Conversely, there has been an increase during evening-night shift and on fast lanes.

The explanatory variables related to type of vehicle presented in Table 5 show that crashes involving motorcycles and heavy vehicles (such as buses, trucks, or other large vehicles) tend to result in a higher number of deaths and serious injuries. Additionally, crashes involving bicycles tend to result in a higher percentage of serious injuries and fatalities compared to crashes involving lighter vehicles (like passenger cars, vans, or minibuses). Two-wheel and heavy vehicles are typically found to increase the severity of crashes when compared to cars [3], [24]. When examining the changes between the periods of 2010-2012 and 2017-2019, it is worth highlighting that crashes involving bicycles have substantially decreased the percentage of serious and fatal victims, even though the presence of this type of crash has increased during these two periods.

Table 6 presents explanatory variables related to causes of crashes. Fatalities and serious injuries are more frequent when the crash is caused by speeding, pedestrian behavior and excessive alcohol consumption, in that particular order. Speeding is the most significant risk factor in relation to road safety [25] and results in a higher number of fatalities and serious injuries compared to minor injuries [19], [4]. Although there has been a notable decrease in the occurrence of excessive speeding-related crashes between 2010-2012 and 2017-2019, the severity of these crashes has notably risen.

The variables related to the type of crash and their incidence are presented in Table 7. The table reveals that crashes involving a run-down or a collision with a stationary object (*Shock*) are more likely to result in fatalities or serious injuries. This finding is consistent with previous research such as [11], who found that incidents involving stationary objects were more likely to result in serious injuries compared to those involving moving vehicles. Additionally, crash involving a run-down pose a greater risk of serious injury or death, as they involve two of the most vulnerable road users: pedestrians and cyclists [26], [24]. Attending the evolution of these types of crash between 2010-2012 and 2017-2019, we notice a decrease in the severity of run-down and collision crashes, while it has either increased or remained stable for the other types.

In this study, we have employed binary logistic regression models to determine the associations between the probability of severity outcomes (e.g. fatal/serious injuries versus minor injuries) and all contributory factors [13]. In our case, the response variable is *Severity* with two levels (0: non-serious; 1: fatal and serious). The logistic regression model, expressed in terms of the logit transformation of the *i*-th individual's response probability, $p_{i}$ (e.g. probability of fatal/serious), is a linear function of the vector of explanatory variables, denoted by $x_{i}$:

(1) $$logit(p_{i})=log\left(\frac{p_{i}}{1-p_{i}}\right)=\beta_{0}+\beta_{1}x_{1}+\cdots +\beta_{i}x_{i}+\cdots +\beta_{n}x_{n}\;,$$

where $\beta_{i}$ is the vector of coefficients and i=1,…,n.

## 3 Results

Within this section, we will present and discuss upon the outcomes achieved from applying various logistic regression models to the data that has been previously introduced.

To begin, Table 8 displays the results of a logistic regression model, which were estimated to determine whether the measures implemented from the LRSP help to reduce the severity of traffic crashes in Barcelona from 2010 to 2019. This model was used to model the binary *Severity* variable. Specifically, the severity is assessed based on a dichotomous variable where the 0-valued category (non-serious injury, the reference category in the logistic model) consists of 85,429 cases, and the 1-valued category includes incidents involving 1, 2, 3 or 4 fatalities/serious injuries per crash, which constitute 2,394 cases. For each risk factor, the estimated coefficients, standard error, and p-value statistic are reported. A confidence level of 90% was used in this study to compensate for efficiency reduction, and variables with a p-value greater than 0.1 were kept in the model to facilitate comparison between models. A positive significant coefficient indicates that the presence of the respective factor increases the severity odds ratio (i.e. the probability of causing serious injury or fatalities with respect to the corresponding reference category). Conversely, a negative significant coefficient reduces this odds ratio.

The results shown in Table 8 reveal that several factors are associated with crash severity, which coincide to a large extent with the descriptive summaries provided in Table 3, Table 4, Table 5, Table 6, Table 7. Assuming that the *Period* variable to a large extent reflects the changes introduced by LRSP Plan, the negative coefficients for this variable assesses the reduction of fatalities and serious injuries in traffic crashes during the periods 2013-2016 and 2017-2019 compared to the reference period of 2010-2012, controlling for all other risk factors included in the model. Specifically, the risk of fatal or serious injuries is lower during the period 2013-2016, with a severity odds ratio reduction of 10%. Furthermore, the risk is even lower during the period 2017-2019, with a severity odds ratio reduction of 18%. Therefore, we can conclude that the measures taken have had a positive effect in reducing the severity of traffic crashes.

**Table 3:** Description of variables.

Name	Description and Levels
*Crash severity*	
Severity	(1: Fatal/Serious victims, 0: Non-serious victims)
*Time of the crash*	
Period	Period in which the crash occurred
	(2010-12, 2013-16, 2017-19)
Daytype	Type of day in which the crash occurred
	(Working day, Weekend day)
Daypart	Part of the day in which the crash occurred
	(Morning, Afternoon, Evening-Night)
*Site of the crash*	
Via	Type of road on which the crash occurred
	(Street, Avenue, Fast lane)
*Vehicle-related characteristics*	
Vehicles	(1: More than one vehicle involved, 0: Other)
Bicycle	(1: Presence of this level, 0: No presence)
Twowheel	(1: Presence of this level, 0: No presence)
Heavy	(1: Presence of this level, 0: No presence)
Light	(1: Presence of this level, 0: No presence)
*Related causes of the crash*	
Pedestrian	(1: Presence of this level, 0: No presence)
Bloodalcohol	(1: Presence of this level, 0: No presence)
Speed	(1: Presence of this level, 0: No presence)
Roadcondition	(1: Presence of this level, 0: No presence)
*Type of crash*	
Rundown	(1: Presence of this level, 0: No presence)
Twowheelfall	(1: Presence of this level, 0: No presence)
Collision	(1: Presence of this level, 0: No presence)
Shock	(1: Presence of this level, 0: No presence)

**Table 4:** Summary of explanatory variables: Daytype, Daypart, Via and Vehicles. Percentages of frequency and severity levels of 2010-2012 period (versus 2017-2019).

Variable	Level	frequency		non-serious	serious	fatalities
Daytype	Working	82.35 (81.23)		97.07 (97.75)	2.56 (2.08)	0.37 (0.17)
	Weekend	17.65 (18.77)		96.29 (96.66)	3.27 (3.04)	0.44 (0.31)

Daypart	Morning	39.61 (39.41)		97.31 (98.10)	2.30 (1.74)	0.41 (0.16)
	Afternoon	49.85 (50.11)		96.84 (97.47)	2.84 (2.39)	0.32 (0.14)
	Even.-Night	10.54 (10.48)		96.00 (95.80)	3.45 (3.58)	0.54 (0.62)

Via	Street	39.48 (32.75)		96.86 (97.59)	2.68 (2.27)	0.46 (0.14)
	Avenue	52.65 (58.21)		96.84 (97.56)	2.82 (2.26)	0.34 (0.18)
	Fast lane	7.87 (9.04)		97.98 (97.25)	1.77 (2.24)	0.26 (0.52)

Vehicles	1 (>1 vehicle)	79.31 (80.11)		97.59 (98.08)	2.19 (1.80)	0.22 (0.12)
	0 (other)	20.69 (19.89)		94.43 (95.39)	4.56 (4.10)	1.01 (0.51)

**Table 5:** Summary of explanatory variables related to types of vehicle involved in the crash. Percentages of frequency and severity levels of 2010-2012 period (versus 2017-2019).

Variable	Level	frequency		non-serious	serious	fatalities
Bicycle	Presence	6.01 (7.98)		97.21 (97.69)	2.38 (2.22)	0.41 (0.09)
	No Pres.	93.99 (92.02)		96.92 (97.53)	2.70 (2.26)	0.38 (0.21)

Twowheel	Presence	65.41 (65.15)		96.72 (97.40)	2.89 (2.42)	0.39 (0.21)
	No Pres.	34.59 (34.85)		97.36 (97.87)	2.29 (1.96)	0.35 (0.18)

Heavy	Presence	6.70 (7.37)		95.12 (96.53)	4.02 (2.79)	0.85 (0.68)
	No Pres.	93.30 (92.63)		97.07 (97.62)	2.58 (2.22)	0.35 (0.16)

Light	Presence	80.25 (76.72)		97.42 (97.86)	2.34 (2.03)	0.24 (0.12)
	No Pres.	19.75 (23.28)		94.97 (96.51)	4.08 (3.02)	0.95 (0.46)

**Table 6:** Summary of explanatory variables related to causes of the crash. Percentages of frequency and severity levels of 2010-2012 period (versus 2017-2019).

Variable	Level	frequency		non-serious	serious	fatalities
Pedestrian	Presence	6.57 (6.76)		92.29 (93.22)	6.22 (5.98)	1.49 (0.80)
	No Pres.	93.43 (93.24)		97.26 (97.86)	2.43 (1.99)	0.30 (0.15)

Bloodalcohol	Presence	2.70 (3.10)		96.67 (95.23)	2.72 (4.54)	0.60 (0.23)
	No Pres.	97.30 (96.90)		96.95 (97.62)	2.68 (2.19)	0.37 (0.20)

Speed	Presence	1.21 (0.48)		92.57 (79.70)	4.05 (15.04)	3.38 (5.26)
	No Pres.	98.79 (99.52)		96.99 (97.63)	2.66 (2.20)	0.34 (0.17)

Roadcondition	Presence	1.20 (0.84)		98.63 (98.71)	1.02 (1.29)	0.34 (0.00)
	No Pres.	98.80 (99.16)		96.92 (97.53)	2.70 (2.27)	0.38 (0.20)

**Table 7:** Summary of explanatory variables related to types of crash. Percentages of frequency and severity levels of 2010-2012 period (versus 2017-2019).

Variable	Level	frequency		non-serious	serious	fatalities
Rundown	Presence	13.95 (12.83)		93.08 (94.52)	5.68 (4.87)	1.23 (0.62)
	No Pres.	86.05 (87.17)		97.56 (97.99)	2.19 (1.87)	0.24 (0.14)

Twowheelfall	Presence	13.53 (14.01)		97.46 (97.76)	2.20 (2.03)	0.33 (0.21)
	No Pres.	86.47 (85.99)		96.86 (97.51)	2.76 (2.29)	0.39 (0.20)

Collision	Presence	70.78 (71.85)		97.57 (98.01)	2.21 (1.87)	2.14 (1.87)
	No Pres.	29.22 (28.15)		95.40 (96.35)	3.82 (3.24)	0.78 (0.41)

Shock	Presence	3.82 (3.11)		92.2 (92.93)	5.99 (5.45)	1.39 (1.62)
	No Pres.	96.18 (96.89)		97.11 (97.69)	2.55 (2.16)	0.34 (0.15)

Additionally, the model enables us to distinguish this effect from other risk factors. The likelihood of a fatal or serious crash is lower on working days compared to weekends, and the highest risk is during evening and night shifts. The severity odds ratio is reduced by 25% during working days, while the ratio is increased by 49% during evening and night shifts compared to morning shifts. Research has shown that crashes are more severe during night shift [19], [27], [3]. Studies on the day of the week have yielded mixed results, there are studies that obtain the same result as us [23], [28], and others studies [24] in which the results show that pedestrians fatalities and serious injuries are less likely to occur on weekends.

Setting regular streets as the reference category, there is a higher risk of a fatal/serious injury in fast lanes, with a 28% increase in severity odds ratio. Conversely, the presence of more than one vehicle in a crash reduces the odds ratio by 61% compared to incidents with only one vehicle. These findings are consistent with other studies [20], [19], [15], [16]. In terms of vehicle type, crashes involving two-wheel or heavy vehicles carry a higher risk of fatality or serious injury compared to light vehicles. For instance, crashes involving two-wheel vehicles are 3.5 times more severe than those without. Studies have shown that two-wheel and heavy vehicles generally increase crash severity [3], [24].

When examining factors related to the cause of the crash, excessive speed is found to be the most risky, increasing the likelihood of serious injuries or death even more than pedestrian behavior. If a crash is caused by excessive speed, the severity odds ratio increases by a factor of 3.7. While excessive alcohol consumption is also a risk factor, it is not as significant, likely due to its correlation with speed and night shift driving. Road conditions do not appear to impact negatively on the risk of serious injury or death. Analysis of different crash types reveals that collisions with stationary objects or being struck by a vehicle are the most likely to result in fatalities or serious injuries, with severity odds ratios increasing by about 4 times.

### 3.1 Logistic models for periods

To study the consistency of the measures applied, we divide our data set in subsamples according to the period which crash occurred. Subsequently, we proceed to estimate two binary logistic regression models: one for the reference subperiod of 2010-2012 and another for the final subperiod of 2017-2019. Table 9 reports the results obtained.

To begin with, the primary modification observed between the two periods pertains to the intercept coefficient. Its sustained reduction indicates an overall decline in crash severity. This reduction encompasses both the impact observed for the *Period* variable in Table 8's model and the mitigation of crash severity for certain baseline risk factor levels, such as morning shift or regular streets.

**Table 8:** Results of the logistic regression model designed to explain crash severity.

Variables	Coefficient	St. Error	P-value
Intercept	-5.059	0.134	<0.01
Period_2013-16	-0.102	0.050	0.041
Period_2017-19	-0.198	0.055	<0.01
Daytype_Working	-0.295	0.051	<0.01
Daypart_Afternoon	0.215	0.047	<0.01
Daypart_Even.-Night	0.397	0.070	<0.01
Via_Avenue	0.077	0.045	0.085
Via_Fast lane	0.246	0.087	<0.01
Vehicles_1	-0.947	0.117	<0.01
Bicycle_1	0.773	0.104	<0.01
Twowheel_1	1.249	0.076	<0.01
Heavy_1	1.359	0.093	<0.01
Light_1	0.421	0.079	<0.01
Pedestrian_1	0.393	0.075	<0.01
Bloodalcohol_1	0.157	0.115	0.172
Speed_1	1.314	0.132	<0.01
Roadcondition_1	-0.904	0.342	0.013
Rundown_1	1.491	0.099	<0.01
Twowheelfall_1	0.208	0.085	0.015
Collision_1	0.706	0.108	<0.01
Shock_1	1.477	0.092	<0.01

**Table 9:** Results of the logistic regression models per period.

Variables	Logistic 2010-12			Logistic 2017-19		
	Coef.	S E.	P-val.	Coef.	S E.	P-val.
Intercept	-4.896	0.233	<0.01	-5.633	0.249	<0.01
Daytype_Working	-0.253	0.095	<0.01	-0.332	0.094	<0.01
Daypart_Afternoon	0.207	0.083	0.012	0.298	0.090	<0.01
Daypart_Even.-Night	0.375	0.126	<0.01	0.618	0.126	<0.01
Via_Avenue	0.039	0.079	0.625	0.042	0.087	0.631
Via_Fast lane	-0.152	0.176	0.386	0.379	0.150	0.012
Vehicles_1	-0.740	0.208	<0.01	-1.132	0.226	<0.01
Bicycle_1	0.539	0.197	<0.01	0.828	0.191	<0.01
Twowheel_1	1.116	0.133	<0.01	1.336	0.140	<0.01
Heavy_1	1.252	0.163	<0.01	1.445	0.172	<0.01
Light_1	0.205	0.139	0.140	0.594	0.148	<0.01
Pedestrian_1	0.143	0.135	0.290	0.521	0.144	<0.01
Bloodalcohol_1	-0.182	0.238	0.443	0.467	0.179	<0.01
Speed_1	0.781	0.246	<0.01	2.015	0.247	<0.01
Roadcondition_1	-0.734	0.519	0.158	-0.410	0.595	0.490
Rundown_1	1.619	0.175	<0.01	1.558	0.195	<0.01
Twowheelfall_1	0.165	0.156	0.290	0.317	0.160	0.047
Collision_1	0.745	0.195	<0.01	0.999	0.209	<0.01
Shock_1	1.559	0.164	<0.01	1.502	0.176	<0.01

In the morning shift, when elderly and school-going youth are more likely to be in transit, the continuous implementation of informative and educational sessions promoting safe mobility from the inception of the plan may have had a positive impact (refer to Table 4). The reduction of crash severity on regular streets (also detailed in Table 4) can be attributed to various measures, including the application of corrective actions at crash hotspots; structural nature measures, like the expansion of peaceful areas in the city; improvement of pedestrian crossings and reinforcement of speed controls, particularly in Zone 30 (single-lane roads where the speed limit cannot exceed 30 km/h); and static and dynamic surveillance campaigns at crossings with speed controls.

Beginning with the initial risk factor of the crash day type and considering its evolution, there has been a more significant reduction in crashes resulting in fatal or serious injuries during weekdays compared to weekends. This trend could be attributed to the impact of the road safety training sessions conducted since 2014 in companies, which aim to provide employees who commute to work and those who operate motorcycles, trucks, or vans for work-related purposes with appropriate safety measures. In the year 2014, work-related trips accounted for 36% of the severe crashes that occurred in the city [29].

When it comes to the time of day when crashes occur, the likelihood of an incident being severe is higher in the afternoon and especially at night compared to the morning. This trend has remained relatively consistent over the years for the afternoon shift, but the crash severity in the night shift has varied more. While both the morning and afternoon shifts have seen a similar reduction in crash severity over time (refer to Table 4), indicating that the measures explained above for the morning shift may have been also effective during the afternoon shift; the night shift has experienced a different pattern. During these years, the odds ratio for severe crashes that occurred at night increased significantly compared to the reference category of morning crashes. This increase is due to the large reduction in severity of morning crashes and a corresponding slight increase in severity for night crashes. The higher likelihood of driving at higher speeds due to less traffic and increased alcohol consumption may be contributing factors to the rise of crash severity at night.

Considering the aforementioned consistent reduction in the severity of crashes on regular streets over time (refer to Table 4), it is notable that the crash severity in avenues has also declined in parallel, as evidenced by the corresponding odds ratios. However, there has been a significant increase in crash severity on fast lanes during the same period. It is possible that driving at higher speeds on these roads is contributing to this trend.

Looking at the type of vehicle, there has been an increase over time in the likelihood of severe injuries for victims involved in crashes with the presence of bicycle, two-wheel, heavy, and light vehicles compared to those without such vehicles. Despite attempts to reduce the severity of crashes involving motorcycles and heavy vehicles, such as stricter power controls and implementation of road safety plans, the effectiveness of these measures remains unclear. As outlined in Table 5, these measures have contributed to a reduction in crash severity in these particular cases, although not to the same degree as observed in other types of crashes. Furthermore, the frequency of crashes involving bicycles has increased significantly over the years, which corresponds to a rise in bicycle usage. However, there has been a slight reduction in the probability of serious injuries and a great decline with respect to fatal cases, which aligns with structural changes implemented, including the expansion of bike lanes and the restriction of bicycles on sidewalks narrower than 3 meters wide.

Regarding the variables related to the underlying cause of the crash, there is an increasing trend in the greater risk factors for severe crashes, such as excessive alcohol consumption and inappropriate speed, particularly with regards to the latter. Despite the implementation of controls since 2014 to reduce the rate of crashes caused by excessive alcohol consumption, as well as static and dynamic surveillance campaigns aimed at preventing risky behavior through speed controls, the aforementioned risk factors continue to persist. Nevertheless, upon examining their progression (as depicted in Table 6), distinct variations emerge. To begin, when considering frequency, it appears that the implemented measures may have played a role in decreasing the occurrence of crashes involving excessive speed. However, conversely, they have not proven effective in reducing the proportion of crashes associated with excessive alcohol consumption. In terms of crash severity, these measures have generally shown limited efficacy, except for a marginal decline in fatalities linked to excessive alcohol consumption. It is noteworthy that a significant upsurge in severe injuries and fatalities stemming from excessive speed has been observed.

Leaving aside road conditions (with non-significant coefficients), there has been a rise over time in the likelihood of severe injuries for victims involved in pedestrian-attributed crashes when compared to those not involving pedestrians. It is crucial to emphasize that this increase is a result of a more substantial reduction in the severity of crashes not involving pedestrians. In fact, as Table 6 illustrates, incidents involving pedestrians have shown a slight reduction in crash severity over these years. However, an increase in incidence has been observed among age groups ranging from 20 to 44 years old compared to the youngest and oldest groups [9]. As mentioned previously, these findings may be attributed to the intensified informative campaigns aimed at promoting road safety among elderly and young people.

Concerning the type of crash, particularly focusing on those with a greater impact on crash severity, it is evident that the severity odds ratios of run-downs and shocks have remained stable over the years. Measures such as the improvement of pedestrian crossings and intersection configurations may have contributed to reducing crash severity in these cases to the same extent as in other types of crashes.

In contrast, we can observe a slight increase over time in the likelihood of severe injuries for victims involved in two-wheel fall and collision crashes compared to those without such circumstances. However, a closer look at their evolution (refer to Table 7) reveals that crash severity has indeed decreased slightly for both types of crashes, albeit to a lesser degree than for other types (baseline levels). In this regard, it appears that the campaigns aimed at discouraging risky behavior among drivers, particularly motorcyclists, have not yielded the desired effectiveness.

### 3.2 Logistic models for levels of severity

Previously, we addressed crashes that involved either fatalities or victims with severe injuries. In this section, our objective is to examine whether there are distinctions between these two levels of severity. Firstly, we seek to determine if both outcomes experienced a similar reduction during the analyzed period. Secondly, we aim to evaluate if the risk factors incorporated in the models influence both types of crashes similarly.

The results of modeling the different levels of severity are presented in Table 10. First, in order to compare crashes with fatalities and with serious injuries with respect to crashes with minor injuries, two logistic regression models were fitted. The response variable for both models had a “0” category representing non-serious injury (NS) crashes, but different “1” categories for serious injury (S) and fatal (F) crashes respectively. Alternatively, a logistic regression model was also fitted using a response variable defined with level “0” representing serious injury (S) and level “1” representing fatality (F).

**Table 10:** Results of the logistic regression models by different levels of severity.

Variables	Logistic S|NS			Logistic F|NS			Logistic F|S		
	Coef.	S E.	P-val.	Coef.	S E.	P-val.	Coef.	S E.	P-val.
Intercept	-5.092	0.143	<0.01	-7.809	0.375	<0.01	-2.788	0.399	<0.01
Period_2013-16	-0.089	0.053	0.094	-0.184	0.146	0.207	-0.222	0.160	0.165
Period_2017-19	-0.157	0.057	<0.01	-0.549	0.174	<0.01	-0.560	0.189	<0.01
Daytype_Working	-0.297	0.054	<0.01	-0.278	0.157	0.077	0.041	0.170	0.808
Daypart_Afternoon	0.250	0.049	<0.01	0.122	0.145	0.400	-0.351	0.157	0.025
Daypart_Even.-Night	0.377	0.075	<0.01	0.482	0.190	0.011	0.274	0.202	0.175
Via_Avenue	0.086	0.047	0.069	0.026	0.138	0.850	-0.139	0.150	0.353
Via_Fast lane	0.179	0.094	0.056	0.748	0.225	<0.01	0.567	0.252	0.025
Vehicles_1	-1.018	0.126	<0.01	-0.623	0.315	0.048	0.396	0.335	0.238
Bicycle_1	0.784	0.108	<0.01	0.634	0.350	0.070	0.125	0.374	0.738
Twowheel_1	1.208	0.080	<0.01	1.531	0.237	<0.01	0.392	0.268	0.144
Heavy_1	1.209	0.100	<0.01	2.293	0.247	<0.01	1.083	0.270	<0.01
Light_1	0.467	0.084	<0.01	0.063	0.243	0.795	-0.216	0.253	0.394
Pedestrian_1	0.375	0.080	<0.01	0.492	0.202	0.015	0.165	0.224	0.460
Bloodalcohol_1	0.218	0.121	0.072	-0.351	0.340	0.302	-0.084	0.353	0.812
Speed_1	1.023	0.156	<0.01	2.413	0.244	<0.01	1.412	0.262	<0.01
Roadcondition_1	-0.915	0.362	0.011	-0.941	1.023	0.358	-0.637	1.193	0.593
Rundown_1	1.362	0.108	<0.01	2.299	0.243	<0.01	0.903	0.271	<0.01
Twowheelfall_1	0.221	0.091	0.015	0.050	0.229	0.826	-0.087	0.255	0.734
Collision_1	0.714	0.115	<0.01	0.615	0.289	0.033	-0.248	0.308	0.421
Shock_1	1.321	0.101	<0.01	2.356	0.211	<0.01	1.135	0.230	<0.01

By comparing the coefficients of the first two models for the 2017-2019 period, it is evident that the risk of a severe injury crash compared to one with minor injuries has decreased over time, although not as much as the risk of a fatal crash. In parallel, the corresponding coefficient of the third model indicates a 43% reduction in the probability of a fatal crash relative to a crash with severely injured victims. Therefore, as stated in the introduction, it can be inferred that the LRSP measures have had a more significant impact on reducing fatalities than on reducing severely injured victims.

When considering the impact of risk factors on each of these severity levels, we can see notable differences between them. On the one hand, certain factors have a more significant influence on the severity of crashes resulting in fatalities than those resulting in serious injuries. By comparing the corresponding coefficients of the first two models in Table 10, we can observe that when the incident happens during the night shift, on a fast road, and with speeding, the likelihood of a crash with fatalities compared to a crash with non-serious injuries increases more significantly than in the case of crashes resulting in serious injuries. This larger impact on fatality is also present in crashes involving run-downs or shocks, as well as heavy vehicles. In the third model, which directly compares mortality and serious injuries, these factors' coefficients are statistically significant, except for the night shift, confirming the greater effect on mortality than on serious injuries. For instance, the probability of a crash resulting in fatalities compared to a crash resulting in only serious injuries increases by 1.80 and 4 times when the incident occurs on a fast lane and with speeding, respectively.

On the other hand, there are certain factors that have a larger impact on serious injury crashes than on fatalities, although in smaller numbers. Table 10 highlights that crashes involving a car or a bicycle during the afternoon shift have a more significant positive effect on the number of seriously injured individuals than on fatalities. In contrast, crashes with more than one vehicle involved reduce the number of serious injuries more than fatalities. It is worth noting that only one factor, crashes occurring during the afternoon shift, exhibits a significant coefficient in the third model. Specifically, these crashes show a 30% reduction in the likelihood of fatalities compared to the probability of having only serious injuries, in contrast to crashes that occur during the morning shift.

## 4 Conclusions

This study aims to evaluate the impact of actions implemented by the Local Road Safety Plan (2013-2018) of Barcelona City Council, whose objective it was to reduce fatalities and serious injuries caused by traffic crashes in the city of Barcelona.

In general terms, by fitting several logistic regression models to the traffic data gathered by the local police between 2010 and 2019, we have found that the implementation of most measures between 2013 and 2016 contributed to a reduction in fatal/serious injuries. This decrease was even more pronounced between 2017 and 2019, which coincided with the conclusion of the plan. These findings suggest that some of the measures had a positive impact on reducing the severity of crashes. Furthermore, we have also seen that fatalities were reduced more significantly than severe injuries.

The findings of the study corroborated earlier research on the link between certain risk factors and the likelihood of sustaining severe or deadly injuries in road crashes. However, we have concentrated on the relationship between crash severity and risk factors such as time and location of the incident, vehicle characteristics, causes related to the crash, and the type of crash to evaluate the consistency of the road safety measures implemented.

On the one hand, it has been observed that certain preventive actions such as road safety educational programs for the elderly and school-going youth, and training sessions in companies, have proven to be effective in mitigating the risk of serious crashes during daytime hours and on workdays. Additionally, the adoption of corrective measures with a structural nature, such as the creation of calm zones, enhancements to pedestrian crossings, reinforcement of speed controls in Zone 30 and on single-lane roads, and the expansion of bicycle lanes, may have resulted in a reduction in the severity of crashes: on regular streets (and to a lesser extent avenues), involving bicycles, and caused by run-down incidents.

On the other hand, we have come across certain measures that appear to be ineffective or whose impact is uncertain. For instance, despite targeted control campaigns for motorcycles and heavy vehicles, the likelihood of severe crashes involving these vehicles remains high compared to those not involving them, and furthermore, this likelihood has continued to rise over the years. Likewise, the likelihood of serious crashes persists even when measures such as stricter controls to prevent drink-driving and speeding are implemented.

As previously mentioned and based on the findings of the logistic regression models, it can be inferred that the LRSP measures may have resulted in a more significant impact on reducing fatalities than on reducing severely injured victims. Nevertheless, when considering fatalities, the highest risk profile pertains to crashes occurring during the night shift on fast roads, involving speeding and impact (shock). This underscores the need for the Barcelona City Council to persist in reinforcing the measures designed to mitigate such incidents.

To conclude, the LRSP measures implemented in Barcelona have contributed to lessen the severity of traffic crashes over the past decade. Besides that, the study reported here might be extended by employing a spatial regression modeling framework to account for spatial autocorrelation and spatial heterogeneity. From a spatial perspective, it would be interesting to analyze the evolution of crash severity across different areas of Barcelona. This perspective should aim at identifying potential disparities between different areas, providing insight into the effectiveness of measures implemented by the LRSP.

## Additional information

No additional information is available for this paper.

## CRediT authorship contribution statement

**Lluís Bermúdez:** Writing – review & editing, Writing – original draft, Visualization, Validation, Supervision, Software, Resources, Project administration, Methodology, Investigation, Funding acquisition, Formal analysis, Data curation, Conceptualization. **Isabel Morillo:** Writing – review & editing, Writing – original draft, Visualization, Validation, Supervision, Software, Resources, Project administration, Methodology, Investigation, Funding acquisition, Formal analysis, Data curation, Conceptualization.

## Declaration of Competing Interest

The authors declare that they have no known competing financial interests or personal relationships that could have appeared to influence the work reported in this paper.

## Data Availability

All data generated or analyzed during this study are available in the Open Data Barcelona platform https://opendata-ajuntament.barcelona.cat/en/.
